# A hybrid hierarchical strategy for registration of 7T TOF-MRI to 7T PC-MRI intracranial vessel data

**DOI:** 10.1007/s11548-023-02836-y

**Published:** 2023-01-20

**Authors:** Lena Spitz, Franziska Gaidzik, Daniel Stucht, Hendrik Mattern, Bernhard Preim, Sylvia Saalfeld

**Affiliations:** 1 Department of Simulation and Graphics, Otto-von-Guericke University (OvGU), Magdeburg, Germany; 2 Forschungscampus STIMULATE, Magdeburg, Germany; 3 Institute of Fluid Dynamics and Thermodynamics, OvGU, Magdeburg, Germany; 4 Department of Biomedical Magnetic Resonance, OvGU, Magdeburg, Germany

**Keywords:** Phase-contrast MRI, Time-of-flight MRI, Vascular co-registration, Vessel centerline

## Abstract

**Purpose:**

7T time-of-flight (TOF) MRI provides high resolution for the evaluation of cerebrovascular vessels and pathologies. In combination with 4D flow fields acquired with phase-contrast (PC) MRI, hemodynamic information can be extracted to enhance the analysis by providing direct measurements in the larger arteries or patient-specific boundary conditions. Hence, a registration between both modalities is required.

**Methods:**

To combine TOF and PC-MRI data, we developed a hybrid registration approach. Vessels and their centerlines are segmented from the TOF data. The centerline is fit to the intensity ridges of the lower resolved PC-MRI data, which provides temporal information. We used a metric that utilizes a scaled sum of weighted intensities and gradients on the normal plane. The registration is then guided by decoupled local affine transformations. It is applied hierarchically following the branching order of the vessel tree.

**Results:**

A landmark validation over Monte Carlo simulations yielded an average mean squared error of 184.73 mm and an average Hausdorff distance of 15.20 mm. The hierarchical traversal that transforms child vessels with their parents registers even small vessels not detectable in the PC-MRI.

**Conclusion:**

The presented work combines high-resolution tomographic information from 7T TOF-MRI and measured flow data from 4D 7T PC-MRI scan for the arteries of the brain. This enables usage of patient-specific flow parameters for realistic simulations, thus supporting research in areas such as cerebral small vessel disease. Automatization and free deformations can help address the limiting error measures in the future.

## Introduction

In clinical research and practice, multimodal image data or different sequences are required for answering diagnostic and research questions. Depending on the data and artifacts, co-registration can be error-prone and often requires highly adapted algorithms.

One such scenario is the analysis of cerebral blood vessels based on the co-registration of phase-contrast (PC) magnetic resonance imaging (MRI) and time-of-flight (TOF) MRI. While the first contains valuable information about blood flow within the scanned volume, it is limited in resolution [1, 2]. In contrast, TOF-MRI can be of high resolution when scanned with a 7 Tesla (T) MR scanner and can yield a highly detailed 3D vessel model [1, 3]. Registering two such images of a patient enables access to PC-MRI’s hemodynamic information within the high-resolution TOF-MRI model.

Small vessels only visible in higher resolutions are of interest for medical research, e.g., in the case of cerebral small vessel disease (CSVD) or treatment of aneurysms, arteriovenous malformations and explorations of neurovascular implants. CSVD is tied to Alzheimer’s disease, but their exact connection is an active research field with many open questions remaining [3, 4].

PCI-MRI data provides blood flow measurements that computational fluid dynamics (CFD) simulations can utilize, enabling an in-depth analysis of shear stress and pressure values. In order for these simulations to be as realistic as possible, patient-specific boundary conditions and a 3D model of the patient’s vessels are needed [5, 6]. PC-MRI scans offer such patient-specific conditions, but their resolution is not sufficient to detect small vessels [4]. Although both imaging techniques rely on MRI, artifacts occur and a sophisticated co-registration is required [7].

Since vessels make up only a small part of the entire image, an image-to-image registration approach may focus on irrelevant information [8]. Thus, many strategies use vessel segmentations for feature-to-feature registration instead, and for our goal of blood flow simulation, a segmentation of the detailed TOF-MRI data is provided. However, there is no fully automatic segmentation for PC-MRI data available or for co-registering PC-MRI data to TOF-MRI overall.

We present a hybrid approach for registration, meaning segmented vessels from TOF-MRI shall be registered to the image values of the preprocessed PC-MRI. In this work,

- We developed a co-registration for high-resolution 7T TOF-MRI to 7T PC-MRI intracranial vessel data
- We implemented a hybrid hierarchical strategy based on centerline fit
- Our metric guides the registration with three components that consider a weighted sum of scaled intensities, a penalty based on gradients on the normal plane and manually placed landmarks
- During optimization, parameters are decoupled for local affine transformations
- We include a *representation* of the smallest vessels, which allows them to be registered despite not being recognizable within the PC-MRI data.

This can aid further exploration of blood flow even in smaller vessels that can be critical to analyze for various neurological pathologies.

**Fig. 1:**
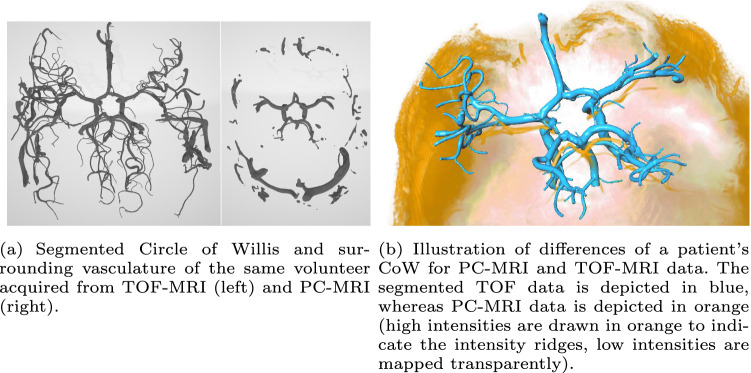
Illustration of TOF and PC-MR images

## Related work

Hybrid co-registration that includes both feature- and image-based information has been used for co-registration of vessels for different modalities. This has the benefit of not requiring as many expensive operations and feature storage as image-to-image or feature-to-feature registrations [9].

In the area of 3D–2D registration, it has been used by Rivest-Hénault et al. [10] for cardiac vessel registration. They extracted the 3D centerlines of the vasculature and registered them, augmenting the images during intervention in real time. Preprocessing included segmentation with a vesselness filter. For registration, a progressively refined affine transformation and a non-rigid method with thin plate splines were used [10].

Other studies employ the entire vessel graph. Aylward et al. [8] performed a centerline segmentation in one image to rigidly register to another image that requires no segmentation. This hybrid approach works under the assumption that vessels in the second image are recognizable as intensity ridges, i.e., they exhibit a large gradient. When the two sets of data are registered, all centerline points lie within these ridges and a weighted sum of scaled intensities metric will be maximized. Results were found to be accurate and robust even for non-rigid deformations and only partial overlap of the images [8].

Other hybrid methods use hierarchical relationships between the vessels by considering the direction of blood flow and parent–child relations. Jomier et al. [11] registered centerlines to intensities hierarchically from root to leaf vessel segments. Furthermore, they use a coarse-to-fine approach and first apply a global rigid, then a local rigid and finally a local non-rigid transformation. To guide the registration, the normal plane perpendicular to each centerline point is calculated, and the surrounding gradients within the vessel radius are projected on it. An iterative optimizer maximizes the intensity sum metric.

Deep learning (DL) methods are becoming more popular for the optimization step of registrations [9, 12]. However, a comparison of iterative optimization and DL reveals that they are highly competitive regarding runtime and accuracy and that iterative methods perform better w.r.t. robustness and parameter changes without needing long supervised training procedures [12].

Powell’s method [13] is a commonly used gradient-free optimizer. It utilizes the concept of conjugate directions and is often extended with Brent’s method, a 1D optimization strategy [14], and performs well in previously mentioned algorithms [10].

## Materials and methods

### Medical image data

A 7T whole-body MRI system (Siemens Healthineers, Erlangen, Germany) was utilized to acquire both sets of MRI data from volunteers. For TOF-MRI, the parameters were set to obtain high-resolution angiograms, which yielded a resolution voxel size ranging from 0.26 to 0.39 mm, while PC-MRI scans ranged from 0.64 to 0.79 mm. We utilized a spoiled gradient echo sequence that featured quantitative flow encoding in all three spatial dimensions [15, 16]. Each time frame had three maps as well as one magnitude image. The velocity encoding value varied among the datasets and was set accordingly to match the highest uniquely resolvable velocity that was assumed by the medical experts [16].

### Extraction of vessels

Both sets of data underwent several preprocessing steps. PC-MRI data were treated according to Bock et al. [17] and then combined into a single dataset by creating a temporal maximum intensity projection of the magnitude image. A vesselness filter, thresholding and masking were applied to both datasets in MeVisLab 3.4.2 [18] to emphasize the vessels. Due to strong variations in vessel size, we found that a combination of multiple vesselness filters worked best [19]. TOF-MRI data were segmented according to [19] via thresholding and masking in MeVisLab as well [20]. After editing in Blender 2.93.4 (The Blender Foundation, Amsterdam, Netherlands), to account for fusion artifacts from the segmentation, the Vascular Modelling Toolkit VMTK 1.4.0 [21] was used to generate centerlines.

The co-registration was implemented in MATLAB R2021a (The MathWorks Inc., Natick, MA, USA). We split the centerline into segments by its branching points, similar to previous work [22]. A graph including parent–child relationships was built, representing the vessel as a tree with root vessels (inlets) that branch out into leaves (outlets). These inherent hierarchical relations were later used for traversal in the optimization strategy.

While the data used included a full Circle of Willis (CoW), it was split at posterior and anterior communicating arteries into three separate vessel trees to eliminate cycles [23].

**Fig. 2:**
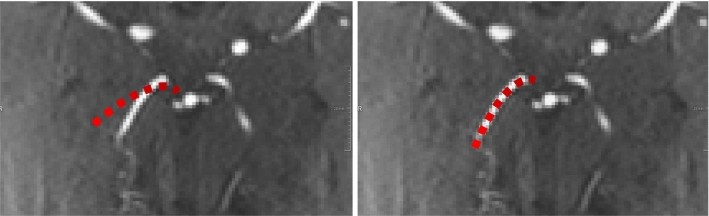
Alignment of red dashed centerline within the intensity ridges. Right picture maximizes the scaled sum of weighted intensities, thus resulting in a good metric, while left picture does not

### Co-registration method

A hierarchical hybrid model–image approach inspired by Aylward et al. [8] was developed for registration, meaning the TOF-MRI centerline was fit to the intensity ridges representing the vessels within the PC-MRI volume (illustrated in Fig. 1, right). We added a hierarchical vessel tree traversal, additional aspects to the metric and a higher-degree deformation.

The registration metric had multiple components. The first component was a scaled sum of weighted intensities, which sums up the intensities of the voxels of the PC-MRI volume in which the TOF-MRI centerline points lie after a transformation step. This sum shall be maximized, so that all centerline points lie within the intensity ridges (Fig. 2, right). The intensities are weighted according to the corresponding vessel radius, derived from the maximum inscribed sphere at the current centerline point. The sum of intensities is scaled based on the weight and amount of centerline points currently considered (see “Coarse-to-fine hierarchical strategy” section). This first part of the metric $m_1(T)$ is thus calculated:

1 $$\begin{aligned} m_1(T) =\frac{1}{\sum _{i=1}^{n}w(r_i)} \sum _{i=1}^{n} w(r_i) I_{\kappa \sigma _i} (x_i T) \end{aligned}$$

where $x_i$ is a centerline point, *T* the transformation, *I* the interpolated intensity value in which the centerline point lies, *n* the number of centerline points and $w(r_i)$ a given point’s weight depending on radius $r_i$ of a given point.

The second component of the metric is a penalty that ensures the centerline points are fit to the middle of the intensity ridge, inspired by Jomier et al. [11]. We extended their approach to prevent that a centerline point is registered to a wrong vessel with a larger or smaller radius by first calculating the perpendicular normal plane at current centerline points within the PC-MRI data. Then, the directions of the gradients on the normal plane within the current vessel radius were examined to see how they correspond to the centerline point position, which ideally would lay within the highest intensity value.

The last component uses landmarks set at vessel branching points to provide additional guidance for vessel start and end points.

### Coarse-to-fine hierarchical strategy

*Tree hierarchy* When finding the right fit of the centerlines to the intensity ridges, the registration strategy made use of a coarse-to-fine approach by utilizing the treelike hierarchy of the vessels [11]. The inlets and outlets are considered roots and leaves, respectively. Vessel bifurcations represent the nodes of the tree. Registration is executed from the roots to the leaves.

Then, the roots’ immediate children are considered the new roots, but remain anchored to their parent vessels during transformation. This keeps the tree together, as well as registering the smallest vessels, which are visible in the TOF-MRI data, but not recognizable in the lower-resolution PC-MRI data. The assumption is that the alignment of children vessels benefits from the alignment of parent vessels.

*Representation concept* In a Boolean variable called *representation*, we record which vessel segments are recognizable in PC-MRI data. This was determined via a vessel segment’s radius, as visibility is determined by vessel size. Thus, represented segments are considered for metric calculation and will eventually become roots for the hierarchical tree traversal. Non-represented vessel segments do not count toward metric calculation and will only be transformed with their parent vessels.

*Multi-start decoupling of parameters* Another aspect of the coarse-to-fine strategy was a multi-start implementation and decoupling of transformation parameters.

**Fig. 3:**
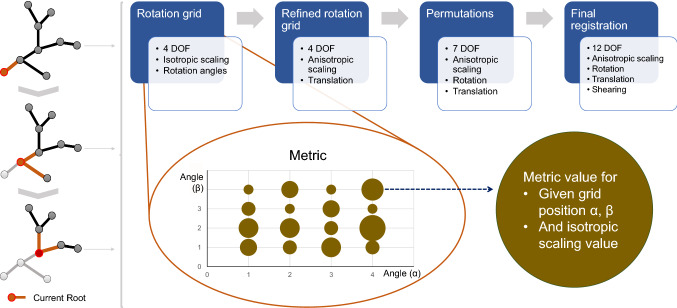
2D illustration of hierarchical top-down strategy and decoupling of parameters: The vessel tree is traversed top-down from root to leaves, each segment becoming the new root and being iteratively registered. Each subtree finds the local affine transformation parameters bottom-up. Rotation angles are resolved via multi-start initialization. Best metric results are taken and refined up to a 12-DOF transformation

Affine transformations were chosen for the bulk of the registration, though a rigid registration is carried out for initialization. The registration steps are based on the observation that rigid transformations are heavily dependent on rotation, which are therefore resolved first [24]. Each registration step goes as follows:

1. Starting with only rotations, a rotation grid is spanned over a set of rotation angles ranging from 0° to 30° in steps of 3° to suit our data. Each point on the grid represents a possible start for the optimization progress.
2. After applying the rotation of one grid point to the data, a 4-degree-of-freedom (4-DOF) optimization is run to find the isotropic scaling and translation parameters. The resulting metric of each point is saved.
3. The grid is then refined through interpolation, inserting a grid point between each two existing ones.
4. From the refined grid, the three best sets of parameters are chosen and every transformation parameter is adjusted using perturbations based on brain radius and voxel size [24], yielding further starting points for a higher 7-DOF optimization.
5. The best set is chosen and will be the starting point for the final 12-DOF optimization featuring three translation, three rotation, three scaling and six shearing parameters (see scheme in Fig. 3).

All steps are repeated for each subtree within the hierarchical vessel tree traversal. For optimization, we used Powell’s method [13] with a step size of 0.1 and a maximum number of iterations of 1000.

### Evaluation

**Fig. 4:**
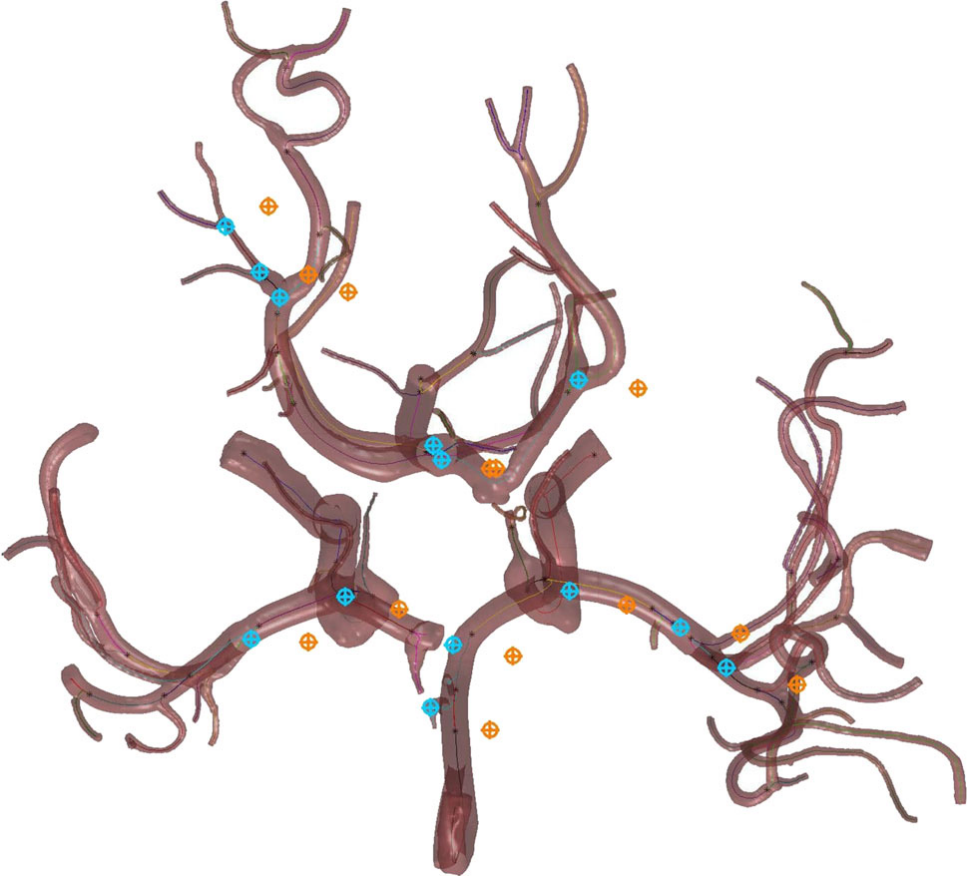
Examples of landmark placement. Orange are PC-MRI landmarks, blue TOF-PC-MRI landmarks

For validation, we considered metric results that shall be maximized, as well as error measures based on landmarks placed at branching points of the vessel trees. Landmark placement was conducted on natural peculiarities (e.g., bifurcations) that could be identified in both sets of data (see Fig. 4). Different sets of landmarks were used for training and validation.

Mean squared error (MSE) and Hausdorff distance (HD) served as error measures, both of which shall be minimized and were recorded before and after registration.

Since 7T MRI is not part of the clinical routine and datasets are rare, we could only use seven paired TOF and PC-MRI scans from healthy volunteers. In order to get a meaningful validation, we artificially transformed them to increase the data size. We opted for Monte Carlo simulations (MCS), i.e., we randomly transformed the TOF-MRI data and registered each of those transformations to their respective (untransformed) PC-MRI data [8, 14].

Strength and number of transformations were adjusted such that the results simulate differences between the original TOF- and PC-MRI data. We chose both rigid and non-rigid transformations to observe our method’s performance for both sets of transformation and due to the discussion of affine transformations being able to solve non-rigid deformations in particular [8].

## Results and discussion

Registration and validation was carried out on a device with an Intel core i7-10850H CPU@2.70G Hz 2.71 GHz processor, 32 GB working RAM and a NVIDIA GeForce RTX 2080 Super graphics card. A single registration took between 20 and 40 min based on size and image quality of the data and complexity of the vessel tree.

**Fig. 5:**
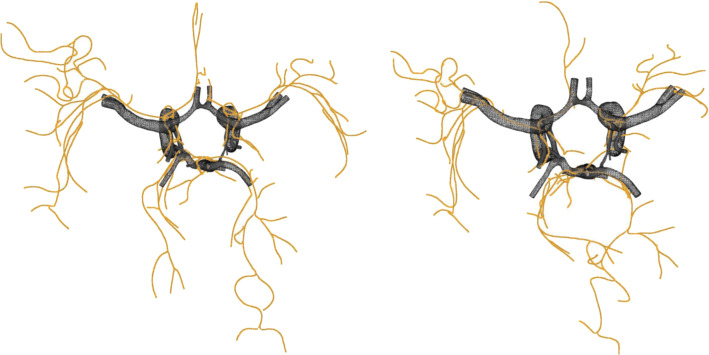
Fit of centerline from TOF-MRI segmentation (orange) to a PC-MRI segmentation done by a clinical expert (black). Left shows preregistration and right post-registration

Before registration, the centerline is scaled too large and does not align with the vessels of the PC-MRI segmentation. After registration, the vessels in the center of the CoW minimize error measures and maximize the metric, though vessels in the periphery seem to be more skewed (see Fig. 5).

Every MCS led to a maximization of the metric, meaning the metric was larger than 0 and had a valid final value. On average, the MSE was reduced from 774.16 to 184.73 mm. There are outliers in the upper ranges, as the median final MSE is 79.21 mm (see Table 1).

**Table 1:** Overall results for MCS

	Metric	Initial MSE	Final MSE	Initial HD	Final HD
Average	3.14	774.16	184.73	31.21	15.20
Median	2.71	664.48	79.21	30.51	13.51
Std. Deviation	2.02	397.88	314.83	9.64	13.68

MSE and HD are given in mm

MSE was minimized in $99.93\%$ and HD was minimized in $99.91\%$ of MCS, meaning that only a very small number of outlier cases registration lead to the error measures, specifically distances between landmarks, worsening rather than improving.

HD was reduced from 31.21 to 15.20 mm on average, with the gap to median values not being as high with an average reduction from 30.51 to 13.51 mm. Standard deviation, however, was 13.68 mm.

There is a trend of non-rigid transformations yielding better registration results, with an average final HD and MSE of 16.91 mm and 221.96 mm for rigid and 13.49 mm and 147.50 mm for non-rigid MCS transformations, respectively (see Tables 2, 3). This is likely due to the rigid transformations leading to a stronger deformation of the data, as the initial HD and MSE is higher for rigid MCS transformations, too.

**Table 2:** Results for rigid MCS

	Metric	Initial MSE	Final MSE	Initial HD	Final HD
Average	3.46	820.88	221.96	33.61	16.91
Median	2.93	780.17	109.22	32.59	15.25
Std. deviation	2.12	438.42	330.16	11.72	13.31

MSE and HD are given in mm

**Table 3:** Results for non-rigid MCS

	Metric	Initial MSE	Final MSE	Initial HD	Final HD
Average	2.83	727.43	147.50	28.82	13.49
Median	2.30	630.20	51.33	28.70	10.84
Std. deviation	1.86	346.46	294.04	6.08	13.83

MSE and HD are given in mm

However, rigid MCS on average minimized their HD to $50.32\%$ and their MSE to $27.03\%$, while non-rigid MCS reduced their HD to $46.81\%$ and their MSE to $20.28\%$. This supports our hypothesis that local affine transformations can approximate the non-rigid deformations in the data that shall be co-registered.

While HD is the maximum distance across all minimum distances of landmark pairs, an average result of 1.5 cm is large for cranial structures. However, the error depends strongly on landmark placement, which was done manually. Even small displacements in landmark placement can lead to higher errors, especially with a measure like MSE.

In comparison, [10] achieved a maximum 3D replacement of 7.416 mm, while [11]’s algorithm registers $87\%$ of the centerline within two voxels of each other, and [8] reached a 0.1–0.2 voxel standard deviation. This shows that while our method reduces the error significantly we have further work to do in our method’s accuracy, for which ideas are presented below. It shall be noted that none of these related works use the exact same data or method.

Registration of non-rigid transformations MCS leads to better results than rigid transformations MCS (see Tables 2,  3). We used local affine transformations for the registration itself, which can only approximate non-rigid deformations. Therefore, replacing them with free deformable transformations could lead to better results.

Since we used MCS by applying transformations to the TOF data, which already has changes to its corresponding PC-MRI scans, we added further transformations and thus made the registration problem harder. However, when registering the seven real-world scans from volunteers, no significant differences are present, other than lower standard deviation (see Table 4).

**Table 4:** Results for original data

	Metric	Initial MSE	Final MSE	Initial HD	Final HD
Average	4.75	563.83	104.67	27.98	16.34
Median	4.21	529.03	97.74	29.78	16.61
Std. deviation	1.84	304.61	57.67	6.41	5.79

MSE and HD are given in mm

The above runtime was measured after acceleration by vectorization. This relates to a preprocessing step, not a step in an interactive process. Nevertheless, additional runtime benefits may be gained by GPU acceleration. Another approach to accelerated runtimes could be a deep learning strategy.

## Conclusion

We presented a hybrid approach to register TOF-MRI data to PC-MRI scans with the goal of combining the high-resolution TOF-MRI segmentation with the blood flow information available in the PC-MRI data. Our registration fits the centerlines of the TOF-MRI data to the intensity ridges of the PC-MRI volume via a weighted sum of scaled intensities metric dependent on radius and vessel normal, and further guidance via landmarks.

We employ information on the recognizability of vessel segments in the TOF data but not in the PC-MRI, yielding a *representation* status. Thus, non-represented segments are treated differently in the registration strategy and are aligned via their parent vessels rather than on their own. Separate landmarks from the ones used to guide registration served for validation purposes.

With an average HD of 15.20 mm and a MSE of 184.73 mm, there is room for improvement. Future work will include the addition of deformable transformations and possibly a non-iterative optimizer. Similarly, we aim to improve the runtime by GPU acceleration. Furthermore, we see potential for improvements through optimizing preprocessing by including motion correction in scan and by utilizing the PC-MRI phase data. An automatization of the segmentation could address concerns for user variability.
